# Integrating palliative care into primary healthcare: assessing the impact of capacity building programs for primary health workers in a coastal region of India

**DOI:** 10.1186/s12912-026-04992-3

**Published:** 2026-07-04

**Authors:** Malathi G. Nayak, Radhika R. Pai, Arun Ghoshal, Naveen Salins

**Affiliations:** 1 Community Health Nursing Department, Manipal College of Nursing, Manipal Academy of Higher Education, Manipal, India; 2 Department of Fundamentals of Nursing, Manipal College of Nursing, Manipal Academy of Higher Education, Manipal, India; 3 Department of Palliative Medicine and Supportive Care, Kasturba Medical College, Manipal Academy of Higher Education, Manipal, India

**Keywords:** Palliative care, Primary health care, Capacity building, Community health workers, Nursing education

## Abstract

**Background:**

Palliative care integration into primary healthcare is essential to improving access for patients with life‑limiting illnesses in low‑ and middle‑income countries. Frontline health workers require targeted capacity‑building to strengthen symptom management and community‑based care.

**Purpose:**

To evaluate the effectiveness of a structured capacity‑building program on the knowledge and attitudes of ASHA workers and ANMs/Staff Nurses regarding palliative care.

**Methods:**

A quasi‑experimental design was used among 984 ASHA workers and 335 ANMs/Staff Nurses in southern India. Participants received structured training on symptom assessment, communication, and home‑based palliative care. Pre‑test and post‑test data were collected using validated knowledge and attitude scales. Data were analysed with Wilcoxon Signed‑Rank tests. The TREND (Transparent Reporting of Evaluations with Nonrandomized Designs) checklist has been completed.

**Results:**

ASHA workers demonstrated significant improvement in knowledge (*r* = .714) and attitude (*r* = .312) scores. Likewise, ANMs/Staff Nurses showed large gains in knowledge (*r* = .973) and substantial improvements in attitude (*r* = .682). The proportion of participants with strong knowledge increased from 9% to 35.2% among ASHAs and from 1.2% to 14% among ANMs/Staff Nurses.

**Conclusion:**

Capacity‑building interventions significantly strengthen primary healthcare workers’ competencies in palliative care. Integrating structured palliative care training into national primary healthcare systems enhances awareness and positive attitudes toward palliative care, thereby strengthening their preparedness to provide supportive care.

Patient or Public Contribution: No Patient or Public Contribution was sought for this research.

**Supplementary Information:**

The online version contains supplementary material available at https://doi.org/10.1186/s12912-026-04992-3.

## Abstract (significance)

What does this paper contribute to the wider global clinical community?

• Demonstrates that structured capacity-building programs significantly improve knowledge and attitudes of primary healthcare workers (ASHAs and ANMs/Staff Nurses) regarding palliative care, highlighting the feasibility and impact of targeted training interventions in low-resource settings.

• Provides evidence supporting the integration of palliative care training into national primary healthcare systems to enhance early identification of symptoms requiring palliative care, increase healthcare workers' knowledge and attitudes toward delivering palliative care in rural and underserved areas where palliative care awareness and delivery are limited.

**Supplementary Information:**

The online version contains supplementary material available at https://doi.org/10.1186/s12912-026-04992-3.

## What does this paper contribute to the wider global clinical community?

•Demonstrates that structured capacity-building programs significantly improve knowledge and attitudes of primary healthcare workers (ASHAs and ANMs/Staff Nurses) regarding palliative care, highlighting the feasibility and impact of targeted training interventions in low-resource settings.

•Provides evidence supporting the integration of palliative care training into national primary healthcare systems to enhance early identification of symptoms requiring palliative care, increase healthcare workers' knowledge and attitudes toward delivering palliative care in rural and underserved areas where palliative care awareness and delivery are limited. 

## Introduction

According to WHO estimates, approximately 20 million individuals globally require end-of-life palliative care services [1]. An equal number are thought to require palliative care in the year before death, for a total of approximately 40 million people annually. Approximately 80% of the 20 million individuals who require palliative care at the end of their lives are thought to reside in low- and middle-income nations; 67% of these individuals are old (over 60), while just 6% are children [2]. Over the last four decades, palliative care in India has undergone steady growth and development, from the early hospice movement in the 1980s to the emergence of specialist and subspecialist palliative medicine in the 2020s [3]. In India, there is a considerable need for palliative care, with a rate of 1.5/1000 population (95% CI: 0.9–2.1) and a prevalence of 6.21 individuals/1000 population. For India, estimates suggest that between 7 and 10 million people require such support annually, yet access is available to barely 4%. Especially, elderly individuals showed an increased prevalence of need for palliative care, with a rate of 37.86/1000 population [4]. 

India has an ageing population, and with increasing NCDs, such as cancer, stroke, heart disease, and lung diseases, there is an increasing need for palliative care to address the serious health-related ailments. Countrywide estimates suggest that around 6.2 people per 1,000 need palliative care, with higher rates in rural areas (about 7.7/1,000) and in the southern states than in the northern states [5, 6]. Among the elderly, about 1 in 8 (12.2%) require palliative and supportive care, especially women, the rural Indian population, and those with chronic disease conditions like cancer, lung disease, and stroke [7].

Despite this, access remains inadequate. Fewer than 4% of those who require palliative care truly obtain it, and for progressive cancer patients, there is a significant unmet need, which is projected to be around 98% [8, 9]. Evidence from community studies indicates similar patterns: Rural studies report 2–3.7 people per 1,000 requiring palliative care, with nearly no one receiving proper treatment and minimal or no awareness of the basic value of palliative care [5, 10]. Collectively, the studies indicate a considerable and mounting nationwide need, with amenities concentrated in a limited number of states and municipal hubs, and foremost gaps in awareness, personnel, medications, and rural access [9, 11]. 

Integrating palliative care into primary healthcare and society is supported as cost-effective, increases the stability of care, lowers cost-sharing for end-of-life care, and aligns care with patients’ preferences to continue at home [12, 13]. The National Program for Palliative Care (NPPC) was launched in 2012 and primarily aimed to deliver accessible, affordable palliative care, integrate it into undergraduate medical education, and encourage public responsiveness and ownership [14]. Though essential services are still not fully incorporated among all levels of the health system, and only a few states have their own palliative care policies [14, 15]. A systematic review reported that community-based care, structured models, and hospital-linked initiatives were the 3 most implemented models of Palliative care in India. Hospital-based interventions showed gaps in their implementation strategies; however, the studies reported a scarcity of long-term outcome data, cost-effectiveness data, and evidence of reproducibility in other settings [12]. 

The National Health Policy 2017 clearly approves palliative care as a necessary health service. It requests opportunities for distance and ongoing training to help general specialists provide basic palliative care at the community level [16]. More recent position statements and policies (e.g., Ayushman Bharat, IAPSM, and IAPC collaborations) emphasise entrenching palliative care into Health and Wellness Centres and public-based programs to improve Universal Health Coverage and Sustainable Development Goal 3 [14, 17].

A systematic review reported that early identification and addressing symptoms requiring palliative care, through the integration of palliative care into primary healthcare, result in financial and clinical benefits, including the potential to prevent unnecessary hospitalisations and reduce healthcare expenses [18]. Studies from Africa have also shown that integrating palliative care into primary healthcare for older adults with comorbidities results in considerable improvements in symptom control and a reduction in hospitalisations [19]. 

Given that the majority of individuals requiring palliative care prefer to remain at home, it is both a medical and ethical imperative to ensure palliative care is provided within community settings, integrated as a core component of primary health care [20]. A systematic review found a substantial unmet demand for palliative care in India. Subgroup analyses show that the prevalence is disproportionately higher in rural areas and the southern region, among women, and among people aged 60 years and older [4]. Given that palliative care and rehabilitation are integral to the continuum of care, they are essential components of palliative care [21]. The healthcare system is hindered by factors such as resource scarcity, poverty, and limited information about available healthcare services, making the expansion of PC services a significant challenge [22, 23].

Home-based care should be the key component of PC in the country. Recognise the family’s responsibility in caring for those with chronic illnesses. They should receive social assistance and be given the resources they need to cope with the situation [24]. There is a considerable responsibility for developing countries to incorporate the dimension of “palliative care principles” within their healthcare systems. WHO suggests a public health strategy for incorporating PC into health-care systems in a cost-effective manner, ensuring that all people who require it have access to it, which is critical for achieving Universal Health Care [25].

ASHA workers, ANMs, and staff nurses working in the community act as linking partners between secondary and tertiary health care and community-level health care. Their responsibilities include delivering maternal and child health services, maintaining village health records, and promoting national health programs, including vaccinations [26].

The existing literature shows a paucity of data on palliative care learning needs and competencies across all cadres of the health care system. Community health workers, including ASHAs and ANMs, are not aware of the presence of palliative care, leading to a plan for a large-scale project to train the fundamental palliative care need assessment and symptom management for home-based palliative care for cancer patients [27]. A survey found that only 12 of 99 nurses at a hospital in Delhi had minimal prior palliative care education, indicating a scarcity of curriculum-based teaching [28].

To expand the availability and use of PCs, medical officers and health care professionals must be trained, and the public must be educated through public awareness initiatives. According to the authors, implementing a PC plan necessitates effective leadership, effective administration, political support, and integration across all levels of care [29].

The present study aims to address these differences by assessing the effectiveness of a structured capacity-building program for ASHA workers and ANMs. By focusing on southern India, where the prevalence of palliative care needs is higher [4].

This research aims to contribute to the evidence base for scalable, community-based palliative care models. The study’s objectives are to evaluate PHCWs’ (Primary Health Care Workers) knowledge of PC using a structured knowledge questionnaire and to measure PHCWs’ attitudes toward PC using a structured attitude scale.

## Methodology

### Study design

A quasi-experimental design with an evaluative approach was adopted to assess the effectiveness of a structured capacity‑building program in improving palliative care competencies among primary healthcare workers. This design was selected because it facilitates comparison of pre‑ and post‑intervention outcomes in naturalistic community settings where randomisation is not feasible.

## Setting and participants

The study was conducted in Udupi District, Karnataka State of India, comprising the talukas of Udupi, Kundapur, and Karkala. PHCWs (Primary Health Care Workers) are the health care workers, including Auxiliary Nurse Midwives (ANMs), Accredited Social Health Activists (ASHAs), and Staff Nurses, who are employed in a particular health care centre. The proposed study site district included 59 Primary Health Centres (PHCs) and 316 Sub-Centres staffed by nurses, Auxiliary Nurse Midwives (ANMs), and Accredited Social Health Activists (ASHAs). ASHA workers are trained female community health volunteers deployed under India’s Ministry of Health and Family Welfare (MoHFW). Auxiliary Nurse-Midwives are health care workers who serve as the first point of contact in rural and underserved areas. ASHA & ANMs are grassroots health workers in India who conduct home visits and provide care in maternal & child health, immunisation, family welfare services, education & awareness, record maintenance, and disease control. Staff Nurses, on the other hand, are employed in a primary health care centre, sub-centres, and community health centres.

Of 1440 health care workers approached, 984 ASHA workers and 335 ANMs/Staff Nurses were enrolled using total enumerative sampling. All available health workers employed at a particular health care centre who consented to participate were included, yielding a response rate of 91.59%.

### Intervention: capacity-building program

A structured capacity‑building program on palliative care was developed by subject experts and validated by experts in palliative medicine and nursing. Two training modules were prepared.

The one used for ASHA workers was brief, focusing mainly on the basic principles of palliative care and the various symptoms patients experience. Their training lasted a day (7 h.) and focused on equipping them to identify cases requiring palliative care in their respective communities.

The ANMs/Staff Nurses capacity building was conducted for 3 days (21 h.), which mainly included content on principles and definition of palliative care, identification of patients requiring palliative care, symptom assessment and management (pain, dyspnea, nausea/vomiting, wound issues, edema, constipation/diarrhea, anorexia/cachexia, fever), communication skills, ethics and psychosocial considerations, and home‑based care procedures.

The capacity-building program was delivered through interactive lectures, demonstrations, case-based discussions, and structured competency checklists by the palliative care physician and nurses. The capacity-building program was conducted in the university academic setting, and each workshop had a maximum of 30–35 participants per batch. All were given training modules in the local language. ASHA workers were to identify cases, and ANMs/Staff Nurses were trained in symptom management and the referral system.

### Recruitment, consent, and ethical considerations

Administrative approval was obtained from the Directorate of Health and Family Welfare, Government of Karnataka, followed by permission from the District Health Officer and PHC Medical Officers. Eligible participants were briefed about the purpose of the study, procedures, risks, and benefits. Written informed consent was obtained from each participant. All study tools were translated into Kannada and back‑translated to ensure semantic equivalence.

### Data collection instruments

Four tools were used for data collection:

- Demographic proforma to document participant characteristics.
- Structured Knowledge Questionnaire for ASHAs (16 items; MCQ, true/false, and fill‑in‑the‑blank formats).
- Structured Knowledge Questionnaire for ANMs/Staff Nurses (32 MCQs).
- Attitude Scales for ASHAs (10 items) and for ANMs/Staff Nurses (30 items) using a 5‑point Likert scale.

Knowledge and attitude scores were categorised into predefined levels. All tools were validated, and the cumulative content validity index was 0.91 for ASHA workers and 0.96 for ANM/Staff nurses. To assess reliability, the split-half method was used for the knowledge questionnaire, and Cronbach’s alpha was 0.82 and 0.88 for the attitude scale. The scores were *r* = .76 and 0.82 for ASHA workers and ANM/Staff nurses, respectively [19]. 

### Data collection procedure

Pre‑test data were collected before the training sessions began. The intervention was then delivered in scheduled batches across the district. Post‑test assessments were administered after completion of the training. For ASHA workers, who had one day of training, the post-test was completed the same day in the evening; for ANMS and Staff Nurses, it was completed on the 3rd day of their training. Data collection was performed uniformly across all PHCs and Sub-Centres by trained research assistants.

### Statistical analysis

Data were analysed using Jamovi software. Descriptive statistics (frequencies, percentages, means, and standard deviations) were used to summarise demographic variables and outcome measures. Because the Shapiro–Wilk test indicated non‑normal distribution of knowledge and attitude scores, the Wilcoxon Signed‑Rank Test was employed as a non‑parametric alternative to the paired t‑test to compare pre‑ and post‑intervention scores. Significance was established at *p* < .05. Effect sizes (r) were calculated to assess the magnitude of change following the intervention. The TREND (Transparent Reporting of Evaluations with Nonrandomized Designs) checklist has been completed.

## Results

*Participant Characteristics -* ASHA Workers (*n* = 984) - The demographic profile of ASHA workers is presented in Table 1. The mean age of participants was 43.31 ± 7.09 years, with nearly half (48.1%) in the 40–49-year age group. Most were married (94%) and had more than 10 years of work experience (44.2%). The majority (97.25%) identified as Hindu.

**Table 1:** Demographic characteristics of ASHA workers and ANMs/staff nurses

Variable	Category	ASHA Workers (*N* = 984) n (%)	ANMs/Staff Nurses (*N* = 335) n (%)
Age (years)	20–29	10 (1.0)	34 (10.1)
	30–39	310(31.5)	168 (50.1)
	40–49	473 (48.1)	71(21.2)
	50–59	173 (17.6)	59 (17.6)
	60–69	18 (1.8)	3 (0.9)
	Mean ± SD	43.31 ± 7.09	39.5 ± 9.32
Marital Status	Married	925 (94.0)	308 (91.9)
	Divorced	9 (0.91)	-
	Unmarried	6 (0.60)	22 (6.6)
	Widow	44 (4.47)	5 (1.5)
Experience (years)	1–5	348(35.4)	75(22.4)
	6–10	201 (20.4)	87 (26.0)
	> 10	435 (44.2)	60 (17.9)
	16–20	-	37 (11.0)
	> 20	-	
Religion	Hindu	957 (97.25)	305 (91.0)
	Christian	14 (1.42)	28 (8.4)
	Muslim	13 (1.32)	2 (0.6)
Designation	Staff Nurse	**-**	195 (58.2)
	ANMs	-	140 (41.8)

Participant demographic Characteristics - ASHA Workers (*n* = 984) - The demographic profile of ASHA workers is presented in Table 1. The mean age of participants was 43.31 ± 7.09 years, with nearly half (48.1%) in the 40–49-year age group. Most were married (94%) and had more than 10 years of work experience (44.2%). The majority (97.25%) were identified as Hindu. Regarding ANMs/Staff Nurses (*n* = 335), the mean age was 39.5 ± 9.32 years, with half (50.1%) aged 30–39 years. Most participants were married (91.9%), and over half (56%) reported having 6–10 years of professional experience.

**Table 2:** Comparison of knowledge scores among ASHA workers before and after the training (*N* = 984)

Knowledge Level	Pre-test f (%)	Post-test f (%)	Statistic	*p*-Value	Effect size
Good	89 (9.0)	346 (35.2)	346,017	< 0.001	0.714
Average	582 (59.1)	536 (54.5)			
Poor	313 (31.8)	102 (10.4)			

Table 2 shows a statistically significant improvement from pretest to posttest (W = 346017, *p* < .001), with a large effect size (*r* = .714), indicating the impact of the capacity-building program. Those with good knowledge increased from 9% to 35.2%, while poor knowledge declined from 31.8% to 10.4%. The proportion of ASHA workers with good knowledge increased markedly from 9.0% (*n* = 89) before training to 35.2% (*n* = 346) after training.

### Attitude outcomes among ASHA workers

**Table 3:** Attitude levels of ASHA workers before and after training (*N* = 984)

Comparison	Pre-test f (%)	Post-test f (%)	Statistic	*p*-Value	Effect size
Positive Attitude	929 (94.4)	954 (97.0)	269,498	< 0.001	0.312
Negative Attitude	55 (5.6)	30 (3.0)			

Table 3 shows that the Wilcoxon Signed-Rank test indicated a significant difference between pre- and post-attitude scores of ASHA workers (W = 269498, *p* < .001, *r* = .312). The proportion of ASHA workers with a positive attitude increased from 94.4% (929) to 97.0% (954) after the training. Similarly, the proportion with a negative attitude decreased from 5.6% (55) to 3.0% (30). These changes suggest that the intervention was effective in enhancing favourable attitudes and reducing unfavourable attitudes among participants.

**Table 4:** Comparison of knowledge scores among ANMs/staff nurses before and after training (*N* = 335)

Knowledge Level	Pre-test f (%)	Post-test f (%)	Statistic	*p*-Value	Effect size
Good	4 (1.2)	47 (14.0)	51,620	< 0.001	0.973
Average	108 (32.2)	260 (77.6)			
Poor	223 (66.6)	28 (8.4)			
Positive Attitude	323 (96.4)	333 (99.4)	44,819	< 0.001	0.682
Negative Attitude	12 (3.6)	2 (0.6)			

Table 4 shows that knowledge scores of ANMs/SNs improved following the training. Before training, most participants (66.6%) had poor knowledge, while only 1.2% demonstrated good knowledge. After training, the proportion with poor knowledge decreased markedly to 8.4%, while the proportion with good knowledge increased to 14.0%. The Wilcoxon Signed-Rank Test demonstrated a significant increase (W = 51620, *p* < .001), with a very large effect size (*r* = .973), suggesting that the training intervention had a very strong impact on participants’ knowledge levels. Overall, these results demonstrate that the capacity-building program was highly effective in enhancing ANMs’ and Staff Nurses’ knowledge.

Following training, a significant improvement in attitude was observed among ANM/SNs (Test Statistic = 44819, *p* < .001). The large effect size (*r* = .682) indicates a strong impact of the training on participants’ attitudes.

Positive attitudes increased from 96.4% to 99.4%, while negative attitudes decreased from 3.6% to 0.6%, demonstrating that the capacity-building program effectively promoted positive attitudes.

## Discussion

The present study demonstrates that a structured capacity‑building program significantly improved the knowledge and attitudes of ASHA workers and ANMs/Staff Nurses regarding palliative care. The large effect sizes observed across groups indicate that even short, targeted educational interventions can substantially enhance frontline health care workers’ competencies in symptom identification, increased awareness, and a positive attitude toward integrating palliative care into communities and underprivileged health sectors where palliative care implementation is inadequate. These findings support the growing evidence that training non‑specialist health workers is both feasible and impactful in low‑resource settings.

The improvement in knowledge among ASHAs and ANMs is consistent with the existing literature, which underscores the value of structured palliative care training. Earlier studies from India and other LMICs have shown that brief, skills‑oriented programs can enhance health workers’ understanding of pain management, ethical aspects of care, and end‑of‑life communication [30, 31]. 

Our findings reinforce this evidence by demonstrating measurable gains among two crucial cadres within India’s primary health system. The substantial improvement among ANMs/Staff Nurses, who entered the training with lower baseline knowledge, reflects the high effectiveness of the intervention in bridging critical skill gaps. Given their professional roles, which primarily involved patient identification, especially after the coronavirus pandemic and prior exposure to patient care needs in community health programs, there is a possibility of a ceiling effect, suggesting that they already held positive attitudes towards palliative care implementation. The positive shift in attitudes toward palliative care observed after training also aligns with prior research, which shows that attitudes strongly influence healthcare providers’ willingness to engage in end‑of‑life care [32]. Attitudinal change is particularly important in the Indian context, where misconceptions about palliative care and fear of opioid use often hinder early referral and community‑based management. The high proportion of participants expressing positive attitudes post‑intervention suggests that such training can contribute to cultural and behavioural shifts within primary health teams. The results also complement work by Nayak et al. (2022) and Pai et al. (2023), who noted that capacity‑building initiatives are essential for strengthening primary palliative care in India [13, 27]. 

Given the high burden of advanced illness in the community, the integration of palliative care into PHC requires a well‑trained workforce capable of providing early symptom relief, supporting families, and coordinating referrals. The effectiveness demonstrated in this study suggests that incorporating palliative care into in‑service training for ASHAs and ANMs could accelerate India’s progress toward universal palliative care coverage.

Our findings should also be viewed within the broader framework of the WHO public health model for palliative care, which emphasises education of healthcare providers as one of its four strategic pillars. By equipping frontline workers with fundamental competencies, the intervention directly addresses system‑level gaps identified in earlier evaluations of palliative care provision in India—particularly the shortage of trained providers at the community level and limited access to home‑based care [22, 33]. The improvements observed in this study thus strengthen the primary care foundation, which is essential for the scalable and equitable delivery of palliative care.

Despite these strengths, several challenges remain. Knowledge gains alone do not guarantee sustained practice change, especially in resource‑limited settings where workload, supply shortages, and structural barriers may limit translation into clinical action. Long‑term follow‑up is necessary to determine whether the observed improvements persist and lead to improved patient outcomes. Additionally, while ASHAs and ANMs demonstrated significant gains, future interventions may need to integrate supportive supervision, refresher training, and digital tools to maintain competency over time.

Finally, this study highlights important implications for nursing practice and policy. Integrating structured palliative care modules into existing training curricula for community health workers and nurses could strengthen India’s primary care system and reduce reliance on hospitals for symptom management. Policymakers should consider scaling the program across districts, with support from the National Health Mission, to address the documented unmet need for palliative care in rural areas. For nursing professionals, this underscores the central role of education, mentorship, and leadership in building a compassionate and competent community‑based palliative care workforce.

## Conclusion

This study demonstrates that a structured capacity-building program significantly enhances ASHA workers’ and ANMs’/Staff Nurses’ knowledge and attitudes toward palliative care. After the intervention, both groups showed improvements in recognising symptoms, understanding core principles, and providing supportive care within communities. These findings emphasise the importance of targeted training to enable frontline workers to deliver timely, compassionate care for those with life-limiting conditions. Given the high unmet palliative care needs in India, especially in rural regions, strengthening primary care is essential for early symptom management and reducing hospitalisations. Incorporating palliative care modules into ongoing professional development for ASHAs and ANMs can promote sustainable community-based care. Health systems might also consider formalising such training within existing public health programs to ensure consistent skill development and reinforcement. While this study confirms the effectiveness of training, future research should explore long-term retention, practical application, and impacts on patients and caregivers. Continuous evaluation will help identify best practices for scaling palliative care education. Ultimately, building a confident, well-prepared nursing and community health workforce is crucial for providing equitable, person-centred palliative care at the community level.

## Supplementary Information

Below is the link to the electronic supplementary material.

Supplementary Material 1

## Data Availability

The datasets generated and analyzed during the current study are available from the corresponding author upon reasonable request.

## Abbreviations

PHCW: Primary health care worker
ASHA: Accredited social health activist
ANM: Auxiliary nurse midwife
PHC: Primary health centre
PC: Palliative care
LMICs: Low- and middle-income countries
WHO: World health organisation
SD: Standard deviation
