# Supplementary material for: Integrating palliative care into primary healthcare: assessing the impact of capacity building programs for primary health workers in a coastal region of India
Source: BMC Nurs. 2026 Jul 4;25:886. doi: 10.1186/s12912-026-04992-3 (PMC13613683; doi:10.1186/s12912-026-04992-3)
Supplement: Supplementary file 1 [file 12912_2026_4992_MOESM1_ESM.pdf]

Structured Knowledge Questionnaire for ASHAs

<u>Instructions:</u> Following statements seeks to obtain your knowledge on palliative care, so read the statement carefully and tick (√) mark against the appropriate response in the box provided.

1.) Palliative care is a care given to
   a.) Cancer patients only
   b.) Cure sickness
   c.) Dying patients only
   d.) *Improve quality of life

2.) State whether true or false among the following:
   Palliative care-
   a.) is given only during terminal stages of the disease – **True/False**
   b.) includes family in the care process- **True/False**
   c.) intends to hasten death **-True/False**

3.) Which among the following disease condition that do **NOT** require palliative care?
   a.) Chronic liver disease
   b.) Dementia
   c.) Kidney failure
   d.) *Obesity

4.) In nonverbal pain scale, pain score of 2 to 3 is considered as
   a.) Mild pain
   b.) *Moderate pain
   c.) No pain
   d.) Severe pain

5.) State true or false
   a.) Cold application should be given for 45 minutes –**True/False**
   b.) Distraction method cannot be used to manage pain –**True/False**
   c.) Hot application decreases the blood flow to the affected region **-True/False**
   d.) Pain is not just a sensation but an emotional experience **-True/False**

6.) Which position is comfortable for the patient with dyspnea?
   a.) *Sitting upright
   b.) Supine
   c.) Side lying
   d.) Prone

7.) Which of the following is an example for high fiber diet?
   a.) *Chapatti
   b.) Jaggery
   c.) Lemon juice
   d.) Sugar

8.) Homemade preparation of ORS includes adding______ tea spoon of sugar and __________ tea spoon of salt to _______liter of drinking water

9.) The temperature of water for tepid sponge should be held between

a.) 30° to 32° C

b.) 28° to 32° C

c.) *29° to 32° C

d.) 25° to 28° C

10.) The elevation of body temperature above _______°C is termed as fever

11.) Which among the following disease condition require colostomy?

a.) *Bowel cancer

b.) Cervical cancer

c.) Pancreatic cancer

d.) Renal cancer

12.) Which among the following disease condition that require frequent oral care?

a.) Alzheimer's disease

b.) *Cancer of buccal mucosa

c.) Neurological disease

d.) Rheumatoid arthritis

13.) What is necessary in effective communication?

a.) *Active listening

b.) Giving advice

c.) Providing comfort

d.) Sympathy

14.) Correct sequence of stages of grief

a.) Denial, depression, anger, bargaining, acceptance

b.) Depression, denial, bargaining, acceptance, depression

c.) *Denial, anger, bargaining, depression, acceptance

d.) Denial, anger, bargaining, acceptance, depression.

15.) State true or false

a.) The aim of end of life care is to ensure patients comfort holistically – **True/False**

b.) Meeting religious needs are not necessary during end of life care **-True/False**

c.) Rigor mortis will go away after 12 hours – **True/False**

d.) Psychological support that is provided to the patient is called as bereavement **-True/False**

16.) Excessive accumulation of fluid in the interstitial space is known as **_____**

Q.NO	Answer key	Score
1.	d	1
2a.	False	1
2b.	True	1
2c.	False	1
3.	d	1
4.	b	1
5a.	False	1
5b.	False	1
5c.	False	1
5d.	True	1
6.	a	1
7.	a	1
8.	6 teaspoon of sugar, 1 level teaspoon of salt for 1 litre drinking water	1
9.	c	1
10.	37°C	1
11.	a	1
12.	b	1
13.	a	1
14.	c	1
15a.	True	1
15b.	False	1
15c.	False	1
15d.	False	1
16.	Edema	1
	**Total**	24

SCORING AND INTERPRETATION

CLASSIFICATION

Good knowledge: [score 17 and above (75%)]

Average knowledge: [11 to 16 (50- 74%)]

Poor knowledge: [10 and below (less than 50%)]

Structured Knowledge Questionnaire for ANMs/Staff Nurses

INSTRUCTION: Following statements seeks to obtain your knowledge on palliative care, so read the statement carefully and tick (**v**) mark against the appropriate response in the box provided.

1. The main aim of palliative care is to

   A. Cure pain
   B. Cure illness
   C. Hasten death
   D. *Improve the quality of life

2. Following is **NOT** a priority for the patient receiving palliative care with a life-limiting illness

   A. Obtaining a sense of control
   B. *Prolonging life
   C. Reducing the burden of symptoms
   D. Strengthening the bond between loved ones

3. Maintaining truthfulness and honesty is a component of

   A. Autonomy
   B. Dignity
   C. Justice
   D. *Veracity

4. One fentanyl transdermal patch lasts for

   A. 48 hours
   B. *72 hours
   C. 74 hours
   D. 76 hours

5. Pain that originates from the internal organ is known as

   A. Incidental
   B. Neuropathic
   C. Somatic
   D. *Visceral

6. On a numerical pain rating scale pain score of 4 to 6 represents

A. Intolerable pain
B. *Moderate pain
C. Mild pain
D. Worst possible pain

7. Following is **NOT** an example for strong opioids

A. Fentanyl
B. Morphine
C. Methadone
D. *Tramadol

8. Dyspnea means difficulty in

A. *Breathing
B. Movement
C. Swallowing
D. Sleeping

9. The best position is used to relieve dyspnea is

A. Prone position
B. *Sitting upright position
C. Side-lying position
D. Supine position

10. In the edema pitting scale grade 3+ indicates

A. Area 1.5–2 times greater than normal.
B. *Deep indentation for 5–30 sec with pressure
C. Edema barely detectable
D. Slight indentation with depression

11. As per the Braden scale the score of 16 to 23 indicates

A. *Best prognosis
B. High risk
C. No risk
D. Very high risk

12. To prevent bedsore appropriate nursing care given is.

A. *Back care
B. Bed bath
C. Feeding
D. Oral care

13. In pressure ulcer full thickness tissue loss with exposed bone, tendon, or muscle can be considered as

A. Unstageable pressure ulcer
B. Stage I pressure ulcer
C. Stage II pressure ulcer
D. *Stage III pressure ulcer

14. In wound management, the common solution used to remove the maggots from the wound is

A. Chlorhexidine
B. Sterilium
C. Spirit
D. *Turpentine

15. Abnormal passage or opening between two or more body organs or spaces

A. *Fistula
B. Ostomy
C. Stoma
D. Wound

16. A decrease in the frequency of passage of formed stools is known as

A. *Constipation
B. Diarrhea
C. Intestinal bowel syndrome
D. Intestinal obstruction

17. Which one of the following is the effective drug for constipation?

A. Benadryl
B. Brozodex
C. Glucose
D. *Lactulose

18. Which among the following is **NOT** the content of ORS

A. Glucose
B. *Potassium citrate
C. Sodium citrate
D. Sodium chloride

19. Marked weight loss and muscle wasting is called as

A. Anorexia
B. *Cachexia
C. Dysphagia
D. Muscle atrophy

20. One of the following aspect is used in the assessment of anorexia and cachexia

A. Temperature
B. Level of consciousness
C. Joint stability
D. *Mid arm circumference

21. Temperature of water for the tepid sponge in degree Celsius should be held between

A. 28 ° to 31° C
B. 34 ° to 36° C
C. *29° to 32° C
D. 26°to 30°C

22. For subcutaneous injection the needle should be inserted at the angle of

A. 30 °
B. *45 °
C. 60 °
D. 95°

23. For an adult the most common site for subcutaneous injection is

A. Anterolateral aspect of the upper arm
B. *Deltoid muscle
C. Ventrolateral muscle of the hip
D. Vastus laterals muscle of thigh

24. In nursing delirium scale the score given for mild to moderate illusion/hallucination is

A. *1
B. 2
C. 3
D. 4

25. Core principle of communication skills except

A. Empathy
B. *Negative regard
C. Respect
D. Trust

26. Which one of the following quality is necessary for effective communication?

A. *Active listening
B. Giving advise
C. Providing comfort
D. Showing sympathy

27**.** Persons emotional response to the event of loss is known as

A. Death
B. Fear
C. *Grief
D. Panic

28. The following expression is **NOT** a part of grief

A. Anger
B. Denial
C. Bargaining
D. *Hunger

29. Correct Sequence of Stages of grief is

A. Denial, Depression, anger, bargaining, acceptance
B. Depression, Denial, bargaining, acceptance, depression
C. *Denial, Anger, bargaining, depression, acceptance
D. Denial, Anger, bargaining, acceptance, depression

30. Temporary muscle rigidity that occurs after death is termed as

A. Algor mortis
B. Liver mortis
C. Pallor mortis
D. *Rigor mortis

31. Gurgling type of respirations that occur due to presence of increased secretions in the back of the throat is known as

A. Bradypnea
B. Cheyne stoke respirations
C. *Death rattle
D. Tachypnea

32. Bereavement is

A. The support provided to the family before the death of the patient
B. *The support provided to the family after the death of the patient
C. Providing comfort care to the patient
D. Providing psychological support to the patient

SCORING AND INTERPRETATION

CLASSIFICATION

Good knowledge: [score 26 and above (75%)]

Average knowledge: [18 to 25 (50- 74%)]

Poor knowledge: [17 and below (less than 50%)]

Answer key

QUESTION NUMBER	ANSWER
1.	D
2.	B
3.	D
4.	B
5.	D
6.	B
7.	D
8.	A
9.	B
10.	B
11.	A
12.	A
13.	D
14.	D
15.	A
16.	A
17.	D
18.	B
19.	B
20.	D
21.	C
22.	B
23.	B
24.	A
25.	B
26.	A
27.	C
28.	D
29.	C
30.	D
31.	C
32.	B

Attitude scale for ASHAs

Instructions: Following statements seeks to obtain your attitude towards palliative care, so read the statement carefully and mark against the appropriate response.

Sl.no	Statement	Strongly disagree	Disagree	Neutral	Agree	Strongly agree.
1.	It is difficult to form a close relationship with the dying person and family					
2.	All dying patients who are bedridden are at risk of developing a pressure ulcer					
3.	Oral care is important to prevent oral infection and bad smell					
4.	I believe smoking and alcoholism will not have any effect on wound healing					
5.	I feel discomfort to provide care for patients with nausea and vomiting					
6.	Learning to communicate with patient requiring palliative care is too easy					
7.	I may get infection if I come contact with the person with colostomy, Tracheostomy, CKD and oral problems.					
8.	I don’t require to understand need of palliative care among patients.					
9.	I feel confident to identify problem in caring patients requiring palliative care					
10.	Educating patient and family in meeting palliative care needs of patient and family is utmost important to me.					

Classification

Positive items: (2, 3, 4, 6, 9,10)

Negative items: (1, 5, 7, 8,)

SCORING

Positive attitude score ranges from: (31 to 50)

Negative attitude score ranges from: (10 to 30)

MAXIMUM SCORE = 50

MINIMUM SCORE = 10

Attitude scale for ANMs/Staff Nurses

Instructions: Following statements seeks to obtain your attitude towards palliative care, so read the statement carefully and tick (v) mark against the appropriate response

S.NO.	Statement	Strongly agree	Agree	Neutral	Disagree	Strongly disagree
1.	Palliative care is given only during the end of life.					
2.	Giving care to the dying person is a worthwhile experience.					
3.	I feel verbalizing his /her feelings is beneficial for the chronically sick persons.					
4.	I feel discomfort to provide care for dying patients with nausea and vomiting.					
5.	I feel only antiemetic's will reduce nausea and vomiting in palliative care.					
6.	All dying patients are at risk of developing a pressure ulcer.					
7.	I think it takes a lot of time to cure a pressure ulcer.					
8.	Oral care is important to prevent oral infection.					
9.	In palliative care, oral care should be performed as per the need.					

10.	It is difficult to form a close relationship with the dying person.					
11.	I know very well how to assess the wound.					
12.	I am worried about my lack of ability to perceive wound infection or complication.					
13.	There is no need to give much importance to the dying patients addiction, to pain relieving medication.					
14.	I believe smoking promotes wound healing					
15.	I am confident that I am providing tracheostomy care correctly.					
16.	I may get infection if I come contact with the person with colostomy, Tracheostomy, CKD and oral problems.					
17.	Families need emotional support to accept the behavior changes of the dying person.					
18.	I believe that I must be brave in the face of loss.					
19.	I feel that I will always carry the pain of grief with me.					
20.	Learning communication skills is too easy.					

21.	I don’t feel that I need to learn communication skills.					
22.	I don’t require to understand the need of palliative care among patients.					
23.	Families should maintain as normal an environment as possible for their dying member.					
24.	Care should extend to the family of the dying person.					
25.	The dying person and his or her family should be the in-charge decision-makers.					
26.	The dying person should be given honest answers about their condition.					
27.	I would be uncomfortable if I entered the room of a terminally ill person and found him or her crying.					
28.	Educating families about death and dying is not a non-family caregiver responsibility.					
29.	Palliative care is as important as curative care.					
30.	Some patients can be excluded from the nursing care when they are at the end stage of death.					

CLASSIFICATION

Positive items: (2, 3, 6, 7, 8, 9, 11,15, 17, 18, 20, 23, 24, 25, 26, 29)

Negative items: (1, 4, 5, 10, 12,13, 14,16, 19, 21, 22, 27, 28, 30)

SCORING

Positive attitude score ranges from: (91 to 150)

Negative attitude score ranges from: (30 to 90)

MAXIMUM SCORE = 150

MINIMUM SCORE = 30

Criterion checklist

Attitude scale

Instruction:

The evaluator is requested to go through the content and provide an opinion by marking (✓) against the specific column of the criteria checklist. Your expert opinion and guidance will be highly appreciated.

Item No.	Relevant		Accurate		Appropriate		Remarks
	Agree	Disagree	Agree	Disagree	Agree	Disagree	
1.							
2.							
3.							
4.							
5.							
6.							
7.							
8.							
9.							
10.							
11.							
12.							
13.							
14.							
15.							
16.							
17.							

18.							
19.							
20.							
21.							
22.							
23.							
24.							
25.							
26.							
27.							
28.							
29.							
30.							
31.							
